# Components of respiration and their temperature sensitivity in four reconstructed soils

**DOI:** 10.1038/s41598-022-09918-y

**Published:** 2022-04-12

**Authors:** Na Lei, Huanyuan Wang, Yang Zhang, Tianqing Chen

**Affiliations:** 1 Shaanxi Provincial Land Engineering Construction Group Co., Ltd, Xi’an, China; 2 Institute of Soil and Water Conservation, Northwest A&F University, Yangling Shaanxi, China; 3 Institute of Land Engineering and Technology, Shaanxi Provincial Land Engineering Construction Group Co., Ltd, Xi’an, China

**Keywords:** Climate-change ecology, Climate change

## Abstract

Seasonal changes characteristics in the respiration of four reconstructed soil masses in a barren gravel land were monitored. The results showed that (1) Respiration and heterotrophic respiration of the four reconstructed soils with added meteorite, shale, sand increased gradually with increasing soil temperatures, reaching its maximum in summer and decreasing to its minimum in winter. the average annual respiration of reconstructed soil with sand was 4.87 μmol·m^–2^·s^–1^, which was significantly higher than the other reconstructed soils (*p* < 0.05). (2) The maximum and minimum values of autotrophic respiration for the four reconstructed soils appeared in August 2018 and January 2018, respectively. the proportion of autotrophic respiration to total respiration was 12.5–38.0%, 9.5–42.0%, 7.7–41.2%, and 5.0–39.3% for the soils with reconstituted meteorite, shale, sand, and soft rock, respectively. (3) The relationship between respiration and the temperature of reconstructed soils can be represented by an exponential function. The 90% to 93% changes in reconstructed soils respiration were caused by soil temperature. The temperature sensitivity (Q_10_) of reconstituted soil with added sand was significantly higher than that of the other three reconstituted soils.

## Introduction

Soil respiration is the primary process whereby terrestrial ecosystems release CO_2_ into the atmosphere^1,2^, with the annual release of CO_2_ via this route being more than 10 times that released by the combustion of fossil fuels^3^. The temperature sensitivity of soil respiration is considered the main factor affecting the response of terrestrial ecosystems to global warming and also determines the feedback of soil respiration to atmospheric CO_2_ concentrations^4^. In the context of continuous global warming, research on the temperature sensitivity of soil respiration has been a constant focus of scholars^5,6^, with the mainstream consensus being that soil respiration is particularly sensitive to variations in temperature^7,8^. However, although temperature and moisture are considered the main factors influencing soil respiration^9^, in reality, the rate of soil respiration is a compound effect, reflecting the mutual influence of multiple factors, including temperature, humidity, and organic carbon content, which accordingly contribute to the complexity of the responses of soil respiration to changes in temperature^10^. Moreover, these responses to temperature change are characterized by spatio-temporal variability^11^, which inevitably exacerbates the complexity of research on the temperature sensitivity of soil respiration.

Although soil respiration comprises both autotrophic and heterotrophic respiration, the contributions of these components to temperature sensitivity remain unclear. Thus, to gain a sufficient understanding of the responses of soil respiration to changing temperature, it is necessary to accurately determine the proportionate contributions of autotrophic and heterotrophic respiration to total soil respiration. Given the differing biological and ecological processes involved in the different components of soil respiration, their responses to temperature change will similarly differ^12^, and consequently, dividing soil respiration into different components is considered key to understanding the mechanisms underlying the response soil respiration to temperature change^13^.

The findings of research conducted to date on the temperature sensitivity of soil respiration and its components have tended to be somewhat inconsistent, with some authors contending that the temperature sensitivity of autotrophic respiration is greater than that of heterotrophic respiration^14,15^, whereas others have indicated that heterotrophic respiration makes a greater contribution in this regard^16,17^. Consequently, the precise mechanisms underlying temperature sensitivity have yet to be sufficiently determined.

Soil reconstruction is a process whereby humans, on the premise of respecting the laws of nature, adopt engineering methods, such as replacement, compounding, increase and decrease, and other technical means, to reconstruct soil structures and improve the quality of the land environment. In the reconstruction process, soils that are considered difficult to use or are unusable, such as those from degraded, contaminated, or inefficiently utilized sires, are transformed into soils that are conducive to the survival and reproduction of living organisms. For example, in an area of coal mining subsidence, mechanical rolling and disturbance caused by construction has been found to alter the original structure and profile of the soil, which in turn has modified important environmental factors affecting the rate of soil respiration rate, thereby resulting in a reduction in the soil respiration Q_10_ value^18^. Furthermore, the findings of a study that examined four types of newly structured soil (sandy loess, sandy loess + weathered coal, sandy loess + weathered coal + soft rock, and sandy loess + soft rock) revealed that weathered coal promoted respiration within the newly structured soils, improved the carbon release rate, and altered the diurnal pattern of soil respiration^19^. Compared with natural soils, differences in those in coal gangue filling and reconstruction areas have been found to lead to certain distinctions between the soil respiration processes. During reconstruction, it was established that differences in the thickness of the upper layer of the coal gangue influenced soil surface respiration to varying extents, with the soil carbon sequestration capacity of the 60–100 cm layer being notably most robust, thereby indicating that soils of these depths would be a more suitable thickness of covering soil^20^.

In this study, we examined the properties four reconstructed soils supplemented with meteorite, shale, sand, or soft rock, respectively. Using a soil carbon flux measurement system, our aim was to determine the seasonal changes in soil respiration and its components in these four types of reconstructed soils, along with the temperature sensitivity of soil respiration. We also sought to clarify the respiratory processes and dynamic change mechanisms of soils reconstituted with different materials. This, we hoped, would enable us to gain a more complete understanding of the potential contribution of reconstituted soil respiration in land remediation, to promote the further development of the carbon cycle theory, and to provide a theoretical basis for accurately assessing regional CO_2_ emissions and thus formulating appropriate CO_2_ emission reduction measures. On the basis of our findings, we propose the use of soil respiration to characterize the environmental friendliness of reconstituted soils, which we anticipate will provide a reference for guiding the future selection of suitable materials for soil reconstitution.

## Materials and methods

### Overview of test plots

**Figure 1:**
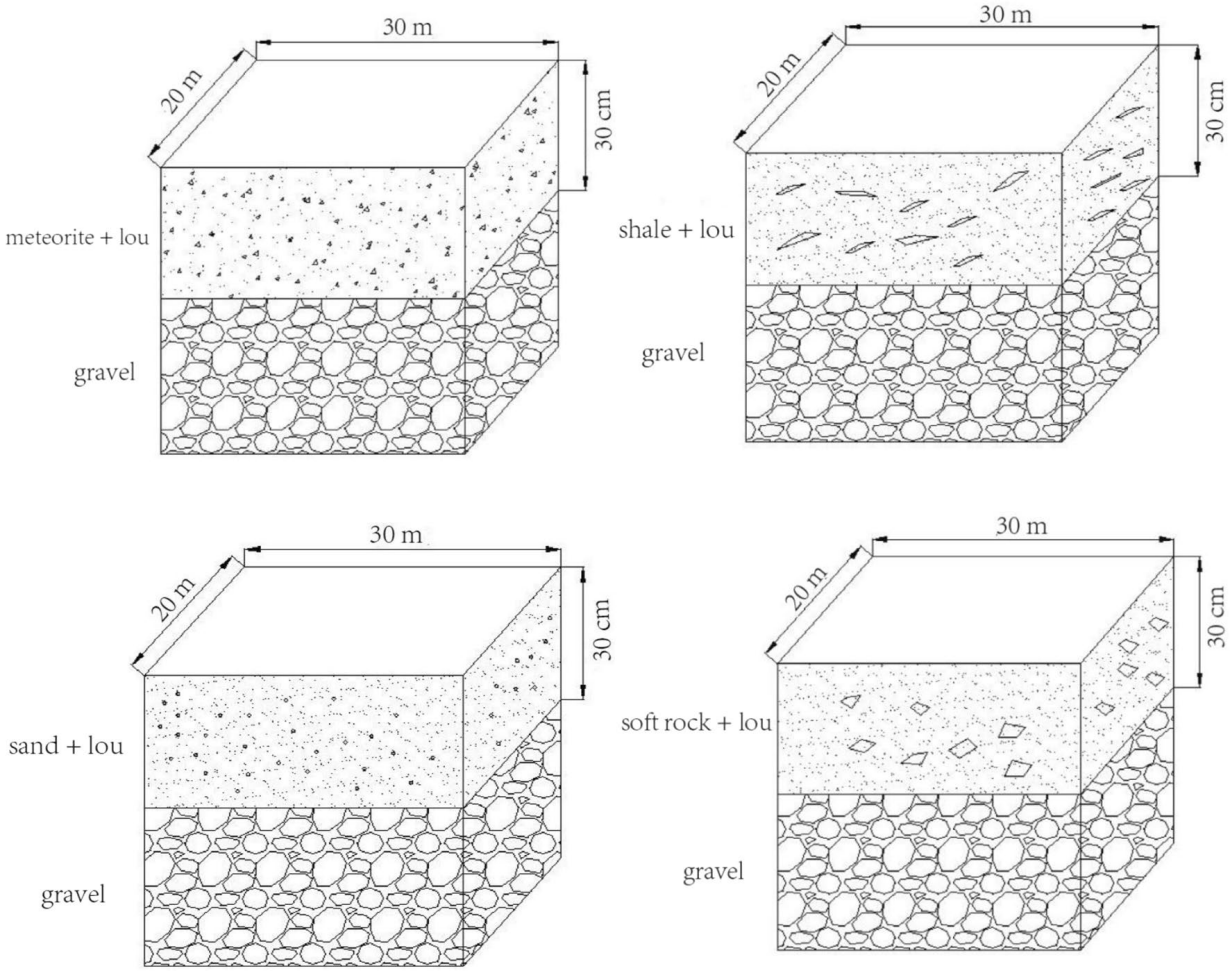
Stereograms of the test sample.

The test plot is located in Shangwang Village, Tangyu Town, Meixian County, Baoji City, Shaanxi Province (107°53′50′′E, 34°8′33′′N), and a demonstration area for the barren gravel land remediation project. The total area is 8.00 hm^2^, and the newly added cultivated land is 6.80 hm^2^. Four materials of soft rock, sand, shale, and meteorite were selected, crushed through a 10 mm sieve, disinfected, sterilized, and mixed with the constructed soil source to form a mixed layer (30 cm) of meteorite, shale, sand and meteorite, and soil. Lou soil, which was the local common soil type, was used for construction. Finally, four reconstituted soils were formed, i.e., gravel + meteorite + lou, gravel + shale + lou, gravel + sand + lou, and gravel + soft rock + lou soil types (hereinafter referred to as meteorite, shale, sand, and soft rock reconstituted soil masses) long-term positioning test^24^ (Fig. 1). The dosage of meteorite, shale, sand, and soft rock was 1 × 10^–3^ m^3^/m^2^. The dimensions of all test plots were 20 × 30 m^2^.

Three soil respiration rings (inner diameter 10 cm) were buried in each of the four test plots, ensuring that the tops of the rings were 2 cm above the ground. At the same time, three small rectangular plots (2 × 2 m)were randomly set up as root exclusion treatment plots. A soil respiration ring of the same specification was buried in each of root exclusion treatment plots. A small trench with a depth of 40 cm was excavated around the root exclusion treatment plots. The excavated ditches were partitioned with as oards, and the soil was backfilled according to the profile level. The vegetation on the ground was cut off in soil respiration rings, ensuring that no vegetation grew in soil respiration rings during the observation period^24^. The physical and chemical properties of the test plots are shown in Table 1.

**Table 1:** Basic physical and chemical properties of four reconstructed soil at 0 ~ 20 cm depth.

Detection indicator		Reconstituted soil mass types			
		Meteorite	Shale	Sand	Soft rock
pH		8.55	8.49	8.51	8.49
Organic carbon (g·kg^-1^)		3.41	3.75	3.7	4.77
Total nitrogen (g·kg^-1^)		0.56	0.36	0.44	0.48
Available phosphorus (mg·kg^-1^)		12.93	26.33	27.27	21.7
Available potassium (mg·kg^-1^)		136.96	130.15	115.54	111.65
Size grading	< 0.002 mm	16.47	16.88	15.17	17.85
	0.002 ~ 0.05 mm	79.87	76.09	79.99	79.22
	> 0.05 mm	6.04	7.03	4.84	2.93

### Research methods

From November 2017 to October 2018, all the soil respiration rings of four test plots were measured on the three typical days each month. The measurement time per typical day was from 9:30 am to 11:00 am, and the time interval was basically 6–8 days. Soil respiration measurements were performed using a soil carbon flux measurement system (LI-8100, LI-COR Biosciences, Lincoln, NE, USA) to measure soil carbon flux, soil temperature at 5 cm and water content at 10 cm. Each soil respiration ring was measured 3 times and the measurement time was 4 min^24^. Autotrophic respiration was obtained by subtraction, that is, soil autotrophic respiration should be the difference between soil total respiration sinus heterotrophic respirations.

### Data analyses

One-way ANOVA was used to analyze differences in soil respiration of the four reconstructed soils. All statistical tests were carried out using SPSS software (version 16.0; SPSS Inc., Chicago, IL, USA). Nonlinear regression was used to assess the relationship between soil respiration and hydrothermal influence factors of the four reconstructed soils, and Q_10_ was estimated. The relationship between soil respiration and soil temperature was fitted by an exponential model (Eq. 1):

1 $$ R_{S} = a{\text{e}}^{bT} ,Q_{10} = e^{10b} $$

where *R*_*S*_ is the soil respiration rate (μmol m^-2^ s^-1^); *T* is the soil temperature (°C); *a* and *b* are the model parameters, and *Q*_*10*_ is the sensitivity coefficient of soil respiration, which refers to the change in entropy of soil respiration rate when the soil temperature rises by 10 °C.

## Results and analysis

### Respiration and heterotrophic respiration of reconstructed soils

We found that the total and heterotrophic respiration of the four assessed reconstructed soils, supplemented with meteorite, shale, sand, and soft rock, respectively, exhibited the same seasonal trends with respect to soil temperature. Specifically, both total and heterotrophic respiration increased gradually in response to increasing soil temperatures, with the trend being highest in summer and lowest in winter. Throughout the entire year, seasonal changes in the total respiration of the meteorite, shale, sand, and soft rock amended soils ranged from 0.16 to 7.97, 0.21 to 9.69, 0.26 to 10.87, and 0.20 to 8.71 μmol·m^-2^·s^-1^, respectively, whereas the corresponding rates of heterotrophic respiration varied from 0.14 to 4.94, 0.19 to 5.62, 0.24 to 6.39, and 0.19 to 5.42 μmol·m^-2^·s^-1^, respectively (Fig. 1). Among the four reconstructed soils, the annual average respiration rate of the soil reconstructed with sand (4.87 μmol·m^-2^·s^-1^) was found to be significantly higher than that of the other three assessed soils (*p* < 0.05), whereas differences among these three soils were shown to be non-significant.

### Autotrophic respiration rate of reconstructed soils and its relationship with heterotrophic respiration

**Figure 2:**
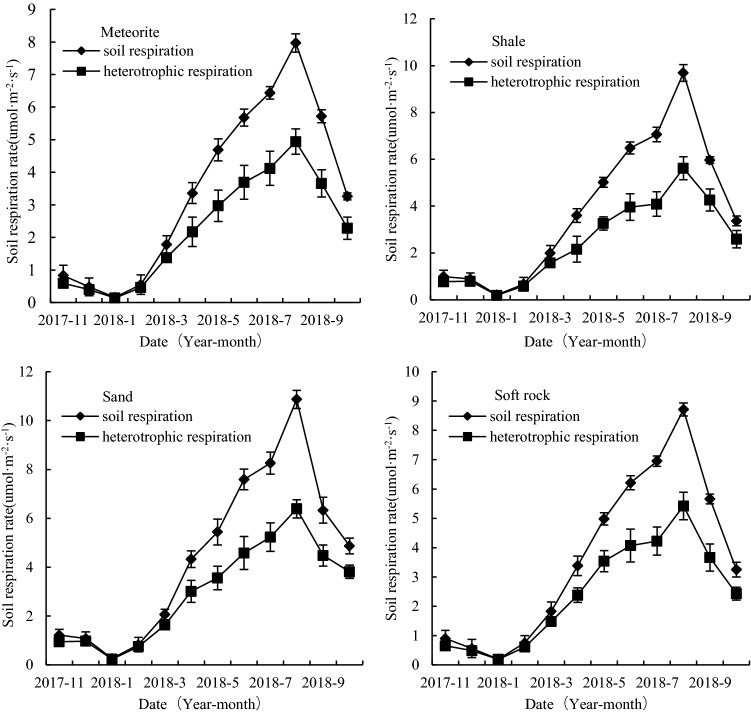
Seasonal changes in respiration and heterotropic respiration of the four reconstructed soils (mean ± standard error).

**Figure 3:**
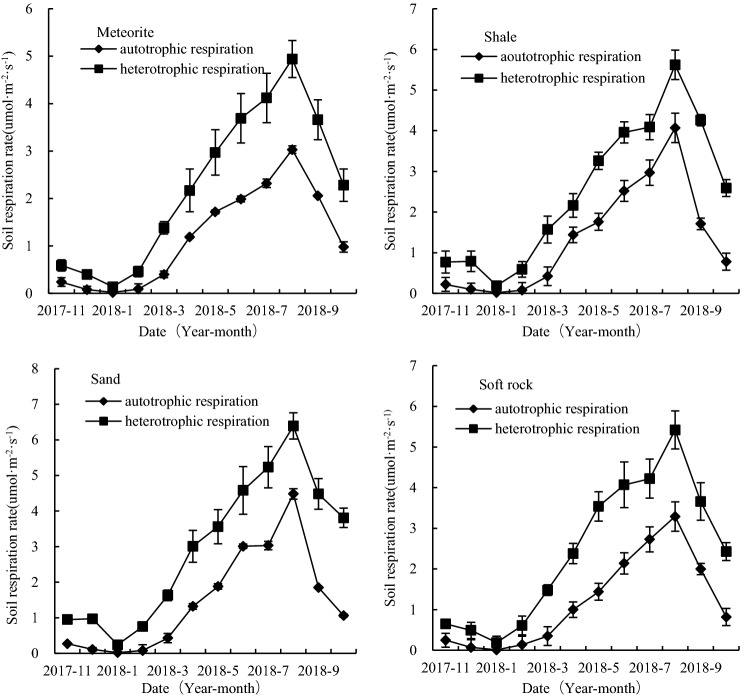
Autotrophic respiration rate of reconstructed soils and its relationship with heterotrophic respiration (mean ± standard error).

The autotrophic respiration of the four reconstructed soils was found to show clear seasonal dynamic changes, with the highest and lowest rates being observed in August 2018 and January 2018, respectively. Among the four reconstructed soils supplemented with meteorite, shale, sand, and soft rock, the highest autotrophic respiration rates were 3.03, 4.07, 3.29, and 5.62 μmol·m^-2^·s^-1^, respectively, whereas the corresponding minimum values were 0.02, 0.02, 0.01, and 0.19 μmol·m^-2^·s^-1^ (Fig. 2). Apart from the summer months, during which we detected significant differences in autotrophic respiration in the meteorite and sand-supplemented soils (p < 0.05), there were no significant differences in the autotrophic respiration of the four reconstructed soils in other seasons (*p* > 0.05). Comparisons of autotrophic and heterotrophic respiration revealed significant differences among the four reconstructed soils in January (*p* < 0.05). During winter, there were significant differences in the autotrophic and heterotrophic respiration of the soft rock-supplemented soil (*p* < 0.05), although during the remaining months, we detected no significant differences in the four reconstructed soils with respect to the two types of respiration (*p* > 0.05) (Fig. 3). Moreover, we established that throughout the entire year, the annual average soil autotrophic respiration rates of the reconstructed soil masses supplemented with meteorite, shale, sand, and soft rock accounted for 12.5–38.0%, 9.5–42.0%, 7.7–41.2%, and 5.0–39.3% of the total soil respiration, respectively (Table 2).

**Table 2:** The ratio of autotrophic respiration to total respiration in reconstructed soils (%).

Year-month	Meteorite	Shale	Sand	Soft rock
2017–11	0.289	0.222	0.221	0.278
2017–12	0.167	0.112	0.102	0.125
2018–1	0.125	0.095	0.077	0.050
2018–2	0.164	0.119	0.096	0.187
2018–3	0.225	0.211	0.209	0.191
2018–4	0.354	0.400	0.305	0.296
2018–5	0.367	0.351	0.346	0.289
2018–6	0.350	0.389	0.397	0.345
2018–7	0.360	0.421	0.367	0.393
2018–8	0.380	0.420	0.412	0.378
2018–9	0.360	0.286	0.292	0.353
2018–10	0.301	0.231	0.218	0.252

### The relationships among soil respiration, soil temperature, and water content

Among the four reconstructed soils, we detected a very significant correlation between the rate of soil respiration and soil temperature (*p* < 0.01), whereas in contrast, there was no obvious correlation between respiration and soil water content. Furthermore, in these soils, the components of soil respiration were found to be significantly correlated with soil temperature (*p* < 0.01) (Table 3), which was identified as the main factor affecting soil respiration. From the perspective of seasonal change, the relationships between soil respiration and soil temperature in the four reconstructed soils can be characterized by exponential functions (Table 4). Among the soils supplemented with meteorite, shale, sand, and soft rock, approximately 90–93% of the changes in soil respiration rate could be attributed changes in soil temperature, with respective Q_10_ values of 3.23, 3.66, 3.89, and 3.50, respectively. Of these values, the Q_10_ of soil reconstructed with sand was found to be significantly higher than that of the other three soils, among which we detected no significant differences.

**Table 3:** Correlation between soil respiration and soil temperature (T), water content (W).

Respiration component	Meteorite		Shale		sand		soft rock	
	*T*	*W*	*T*	*W*	*T*	*W*	*T*	*W*
Total Respiration	0.952**	0.416	0.942**	0.566	0.937**	0.363	0.955**	0.487
heterotrophic respiration	0.960**	0.429	0.942**	0.571	0.944**	0.380	0.971**	0.493
autotrophic respiration	0.934**	0.491	0.913**	0.541	0.899**	0.323	0.914**	0.468

**, *p* < 0.01*, *p* < 0.05.

**Table 4:** Relationship between annual soil respiration (R) rate and temperature (T).

Reconstituted soil mass types	Relationship	Model types	R^2^	Q_10_
Meteorite	*R*&*T*	*R* = 0.3021e^0.1343*T*^	0.90	3.23a
Shale	*R*&*T*	*R* = 0.3593e^0.1242*T*^	0.92	3.66a
Sand	*R*_S_&*T*	*R* = 0.3838e^0.1182*T*^	0.92	3.89b
Soft rock	*R*&*T*	*R* = 0.3194e^0.1227*T*^	0.93	3.50a

## Discussion

### The variation law of soil respiration rate and its components in different reconstructed soils

Among the four reconstructed soils assessed in this study, we detected clear variations in the rates of soil respiration and its components, with high and low values be detected in summer and winter, respectively. These observations are consistent with those previously reported for the seasonal characteristics of reconstructed soil respiration in areas with coal mining subsidence^18^, which have been shown to be determined by temperature and soil moisture conditions^25^. With respect to the different components of soil respiration, organic carbon is primarily released into the atmosphere via heterotrophic respiration, thereby contributing to ecosystem carbon cycling, and thus influencing global climate change^26^. In the present study, we identified consistent trends in the heterotrophic and total respiration of the four reconstructed soils, all of which showed a single-peak curve. These findings are similar to those obtained for the soil respiration characteristics of newly constructed soils in sandy loess dumps located in the Shanxi, Shaanxi, and Mongolian mining areas of China^19^. With respect to autotrophic respiration in the four reconstructed soils, we found that the proportional contribution of this component to the total respiration ranged from 5.0 to 42.0%. Comparatively, a previous study has reported percentage autotrophic respirations ranging from 13 to 94%^27^, and in a further study, the proportion of autotrophic respiration in the soil of a cold zone was found to be 50%–93%, whereas that in a temperate zone was 33–62%^28^. These findings would accordingly tend to indicate that the proportion of autotrophic respiration within soils can be influenced to varying extents by vegetation, time, temperature, and methods of measurement^29^. Given its sources, autotrophic respiration shows clear changes in response to changes in climate, time of day, and season, and predictably, the contribution of autotrophic respiration to total soil respiration will typically be higher during the growing season, and relatively low during the time of year when growth ceases or is substantially reduced^27^.

### The relationship between soil respiration and hydrothermal factors

Although water content and temperature have been established to be the main environmental factors influencing soil respiration in Chinese farmland ecosystems, this respiration and its components are characterized by differential responses to variations in temperature and water content^30^. The findings of numerous studies have indicated that soil temperature is the main factor influencing soil respiration, which is clearly reflected in the observed seasonal changes^31,32^. Consistent with the opinion of a majority of scholars^33,34^, we detected an exponential correlation between soil respiration and soil temperature among the four assessed reconstructed soils. In contrast, in a study examining the CO_2_ flux of reconstituted soil under different ecological restoration modes (vegetation type and covering soil thickness) in the Huainan mining area, the authors concluded that the relationship between respiration and soil water volume can be represented by a quadratic function^35^. Moreover, correlation analyses revealed a non-significant association, with corresponding R^2^ values of between 0.08 and 0.44, which is broadly consistent with our finding for the four reconstructed soils examined in the present study (R^2^ values of between 0.363 and 0.487). Compared with temperature, observed differences in the influence of soil moisture on soil respiration tend to be a little more complex. For example, differences in the total annual precipitation and soil structure of different study sites area may contribute to modifying the relationships between soil moisture and respiration. Furthermore, it can be envisaged that there exists a threshold determining the influence of soil moisture on soil respiration, and that the effect is manifested only when this threshold is exceeded.

The temperature sensitivity of soil respiration varies depending on soil and climatic conditions^36^, and can serve as an important indicator in quantifying and predicting the responses of ecosystems and global carbon cycles to climate change. In China, it has been established that soil respiration Q_10_ values range between 1.09 and 6.27, with an average value of 2.26^37^, and that among different ecosystem types, values follow the order, forest (2.35) > farmland (2.18) > grassland (2.03)^37^. In the present study, we found that respiration in the four reconstructed soils was particularly sensitive to changes in temperature, with corresponding changes in Q_10_ values of between 3.23 and 3.89. The temperature sensitivity of the reconstructed soils was found to be more pronounced than that of farmland ecosystems, particularly after manual intervention. Our findings tend to indicate that the physical and chemical properties of the reconstructed soils and the ecological environment in the study area have, to varying extents, contributed to modifying the gaseous and material circulation processes. In particular, changes in the underlying soil surface have led to changes in soil temperature and moisture, which in turn have influenced respiration within the reconstructed soils.

## Conclusion

1. Respiration within the reconstructed soils and the corresponding carbon emissions were found to be dependent on the materials used to supplement these soils. Our findings indicate that soils reconstructed with meteorite would be beneficial with respect to protection of the ecological environment, whereas soil reconstructed with sand would be unsuitable in this regard.
2. Soil heterotrophic respiration (soil microbial and animal respiration) can be used to represent total soil respiration. In future studies, it will be necessary to examine the contributions of microbial and animal respiration in reconstructed soils to facilitate the development of a better soil mass structure that is ecologically and organically beneficial.
3. When governments implement land remediation plans, if budgets permit, they should prioritize amendment using materials that contribute to environmental protection. Furthermore, carbon dioxide emissions from reconstructed soils should be taken into consideration, thereby enabling the formulation of effective regional measures that are deemed ecologically sound,
